# Safety Evaluation of TiO_2_ Nanoparticle-Based Sunscreen UV Filters on the Development and the Immunological State of the Sea Urchin *Paracentrotus lividus*

**DOI:** 10.3390/nano10112102

**Published:** 2020-10-23

**Authors:** Riccardo Catalano, Jérôme Labille, Daniela Gaglio, Andi Alijagic, Elisabetta Napodano, Danielle Slomberg, Andrea Campos, Annalisa Pinsino

**Affiliations:** 1 Aix Marseille University, CNRS, IRD, INRAE, Coll France, CEREGE, 13545 Aix-en-Provence, France; catalano@cerege.fr (R.C.); labille@cerege.fr (J.L.); Slomberg@cerege.fr (D.S.); 2 Consiglio Nazionale delle Ricerche, Istituto di Bioimmagini e Fisiologia Molecolare (IBFM), 20090 Segrate, MI, Italy; daniela.gaglio@ibfm.cnr.it; 3 SYSBIO.IT, Centre of Systems Biology, University of Milano-Bicocca, 20126 Milano, Italy; elisabetta.napodano@unimib.it; 4 Consiglio Nazionale delle Ricerche, Istituto per la Ricerca e l’Innovazione Biomedica (IRIB), 90146 Palermo, Italy; andialijagic@gmail.com; 5 Aix Marseille Université, CNRS, Centrale Marseille, FSCM, CP2M, 13397 Marseille, France; andrea.campos@univ-amu.fr

**Keywords:** cosmetic formulation, cosmetic lifecycle, hydrophobic compound, nano-TiO_2_, nano safety, marine invertebrate, metabolomics

## Abstract

Sunscreens are emulsions of water and oil that contain filters capable of protecting against the detrimental effects of ultraviolet radiation (UV). The widespread use of cosmetic products based on nanoparticulate UV filters has increased concerns regarding their safety and compatibility with both the environment and human health. In the present work, we evaluated the effects of titanium dioxide nanoparticle (TiO_2_ NP)-based UV filters with three different surface coatings on the development and immunity of the sea urchin, *Paracentrotus lividus*. A wide range of NP concentrations was analyzed, corresponding to different levels of dilution starting from the original cosmetic dispersion. Variations in surface coating, concentration, particle shape, and pre-dispersant medium (i.e., water or oil) influenced the embryonic development without producing a relevant developmental impairment. The most common embryonic abnormalities were related to the skeletal growth and the presence of a few cells, which were presumably involved in the particle uptake. Adult *P. lividus* immune cells exposed to silica-coated TiO_2_ NP-based filters showed a broad metabolic plasticity based on the biosynthesis of metabolites that mediate inflammation, phagocytosis, and antioxidant response. The results presented here highlight the biosafety of the TiO_2_ NP-based UV filters toward sea urchin, and the importance of developing safer-by-design sunscreens.

## 1. Introduction

Ultraviolet (UV) radiation is a main risk factor for skin disorders such as erythema, photoaging, and keratinocyte cancer. As a consequence, effective photoprotection is of utmost importance to humans. Several approaches for skin protection have recently been developed, including organic (e.g., benzophenone, octocrylene) and inorganic UV filters (e.g., zinc oxide, titanium dioxide), topically applied antioxidants, DNA repair enzymes, and oral photoprotective strategies based on nutritional supplements [1]. Sunscreens are emulsions of water and oil that contain UV filters capable of protecting human skin from the detrimental effects of UV radiation [2].

The widespread use of these products has increased concerns regarding their safety and compatibility with both the environment and human health [3]. Thus, sunscreen product-related environmental health risk assessment and management are important issues that need to be carefully considered. Some biological models have shown harmful effects in the presence of organic filters (e.g., benzophenones, camphor) used in sunscreen formulations [4,5,6]. Moreover, most organic UV filters are able to pass through the skin barrier after one single topical application, raising new concerns regarding consumer safety [7]. Thus, inorganic UV filters are becoming even more promising candidates for photoprotection [8]. They consist of either zinc oxide (ZnO) or titanium dioxide (TiO_2_) mineral particles. However, to date, knowledge on the potential health risks associated with sunscreens containing such inorganic UV filters remains limited. ZnO-based UV filters are often less preferred because of their high solubility in water. The dissolved Zn species are more bioavailable than ZnO particles, which increases the potential risk, notably in seawater ecosystems [9,10,11]. On the other hand, TiO_2_-based UV filters are generally considered to be more safe because they are weakly soluble in aqueous media [4,11]. The nanoparticulate form of ZnO and TiO_2_ (size < 100 nm) is generally used in sunscreens because their small size leads to a more transparent film on the skin after application, which enhances user acceptance. Moreover, these metal oxides are also more efficient UV blockers when finely dispersed in the sunscreen product [12,13], which also contributes to the considerable use of nanoparticles (NPs).

However, using NPs also implies specific safety concerns due to their well-known higher surface reactivity and bioavailability [14]. Nanoparticle risk assessment must, therefore, be developed in the context of the sunscreen lifecycle. Although nano-TiO_2_ is one of the most studied nanomaterials in nanosafety research, few of the studied NPs were relevant to the nanoparticulate TiO_2_ UV filters used in sunscreens. In a review of more than 200 scientific articles, Minetto et al. [15] revealed that almost all the safety investigations on TiO_2_ NPs were performed with bare anatase or with the highly photocatalytic P25 (mixture of anatase:rutile, 4:1), whereas the TiO_2_ NPs used as UV filters in sunscreen consist of pure rutile generally coated with a surface passivation layer [16,17]. The aging, transformation, and environmental fate of TiO_2_ NP-based UV filters have scarcely been studied to date [18]. While the important role of the NP coating in controlling fate in the suspension and surface reactivity has already been pointed out, questions as to how its nature and lifetime may influence both exposure and hazard in aquatic systems remain. These are key questions regarding the risk assessment of inorganic UV filters, which cannot be evaluated with the widely studied bare TiO_2_ NPs.

Moreover, the dispersing medium carrying the NPs in the original cosmetic formulation may also play an important role on the environmental fate. Either an oil or water phase can be used at the product fabrication stage to disperse the hydrophobic or hydrophilic TiO_2_ NP-based UV filters, respectively. While the hydrophilic filters pre-dispersed in the water phase can readily be dispersed, diluted, and transported in the aqueous environment, the hydrophobic filters pre-dispersed in the cosmetic oil may have a different environmental fate [19,20]. Once the sunscreen is washed off the skin, the hydrophobic compounds tend to remain in the oil phase of the cosmetic emulsion and float at the water surface. We can reasonably assume that before NP aging takes place, the local NP concentration in the oil droplet floating at the surface should correspond to that in the original cosmetic oil formulation [19]. This is a NP concentration leveling at 5–10 wt%, much higher than any predicted environmental concentration at the ng or µg/L level [21,22]. Nevertheless, organisms living in coastal zones at low water depths could be exposed to these higher NP concentrations. In such an exposure scenario, the oil droplet may act as a vector, transporting the concentrated NP UV filters between the seawater and living organisms, and significantly impacting the biological effects. Currently, there is no reported environmental threshold for TiO_2_ NP-based UV filters inducing a biological effect. It is, thus, mandatory to address the impacts they may have both in the environment and in the organisms.

In the present work, we explored the biosafety of three TiO_2_ NP-based UV filters on the sea urchin (*Paracentrotus lividus*) model both in the embryonic and adult life-cycle stages. Sea urchins, found in almost all the marine environments [23], are an effective sentinel of environmental stress, notably in coastal areas that are potentially more impacted by recreational bathing activity. The sea urchin embryonic development and the immunological state of adult sea urchin immune cells were investigated in culture conditions. A wide range of TiO_2_ NP concentrations was explored, corresponding to different levels of dilution or aging, starting from the original cosmetic dispersion. Considering the contrasted exposure scenarios expected, with an oil phase carrying hydrophobic UV filters and a water phase carrying hydrophilic UV filters, we varied the nature of the TiO_2_ NP coating (hydrophobic: Polydimethylsiloxane and stearic acid; hydrophilic: Silica) and of the cosmetic dispersing medium (water, oil) that were injected into the culture medium. The TiO_2_ NP-based UV filters appear safe, as they did not significantly compromise the growth of the sea urchin embryos at the pluteus stage (from 0.001 to 1 mg/L). The hydrophilic silica-coated TiO_2_ NPs did not affect immune cell viability or produce toxicity at concentrations representative of the cosmetic lifecycle, and the cells showed a broad metabolic plasticity based on the biosynthesis of metabolites promoting an increase in antioxidant activity and phagocytosis.

## 2. Materials and Methods

### 2.1. Commercial TiO_2_ NP-Based UV Filters and Sunscreen Oil Phase

Four different types of TiO_2_ NPs were used in this work. Three of them were commercial nanoparticulate UV filters commonly used in sunscreen formulations, namely, T-S (Titanium Dioxide and Alumina and Stearic Acid), T-Lite (Titanium Dioxide and Aluminum Hydroxide and Dimethicone/Methicone Copolymer), and T-AVO (Titanium Dioxide and Silica). Both T-S and T-Lite UV filters have a primary mineral layer of aluminium oxide and an outermost organic and hydrophobic layer composed of stearic acid or polydimethylsiloxane, respectively. In contrast, the T-AVO UV filter has one single mineral, hydrophilic silica (SiO_2_) coating. In addition, P25 TiO_2_, was used as an uncoated nano-TiO_2_ reference.

These NPs were directly purchased from the suppliers as dry powders. The respective trade names together with the chemical compositions and primary particle size provided by the manufacturers (when available) are reported in Table 1.

Two different dispersing media were used to mimic the release of these NPs and their respective life cycles in the environment. The hydrophobic UV filters (T-S and T-Lite) were pre-dispersed in a typical cosmetic oil, while the hydrophilic NPs (T-AVO and P25) were pre-dispersed in pure water. Further dilution was then completed to reach the desired concentrations, as detailed below.

The cosmetic oil was prepared by mixing together two emollient oils and an emulsifying agent provided by the respective suppliers (see Table 2), in a 2:2:1 ratio. The mixture was gently homogenized by magnetic stirring for 10 min. The emulsifying agent contained two surfactant molecules (Octyldodecyl xyloside and PEG30 dipolyhydroxystearate) known to play a role in the exposure route of the hydrophobic NPs via the creation of oil droplets containing the NPs that are stable in pure water [13].

### 2.2. Sea Urchin Paracentrotus Lividus Embryo Exposure during Development

Six males and six females were induced to spawn by injecting 1–2 mL of 0.1 M KCl into the sea urchin body cavity, through the peristomal membrane surrounding the mouth. Eggs were collected by placing spawning females on 100 mL beakers with 0.45 μm filtered artificial seawater (ASW). Egg quality and sperm motility were inspected by observing the gametes under an optical microscope (OLYMPUS CKX31, Olympus, Tokyo, Japan); 10 μL seminal fluid was added to the egg suspension (sperm/egg ratio 50:1) and fertilization success was verified under the microscope (formation of the fertilization membrane with a fertilization rate >95%). After fertilization, the embryonic culture (500 embryos per mL) was transferred into 50-mL disposable, sterile tubes (10 mL in each tube), and embryos were immediately exposed for 48h to increasing NP concentrations (between 0.001 and 1 mg/L). The hydrophilic P25 TiO_2_ and T-AVO NPs were pre-dispersed and diluted in water in order to reach the desired exposure concentrations. The hydrophobic T-S and T-Lite NPs were pre-dispersed in the oil phase, as typically done during sunscreen formulation. The oil dispersion was then emulsified by dilution in pure water. An oil/water emulsion with a nominal concentration of 5 mL/L was obtained. This emulsion was then added to the embryo culture medium at nominal concentrations of 0.005, 0.05, and 0.5 mg/L. In preparing the different dispersion concentrations, a constant volume of oil was injected into the culture medium, and only the NP concentration was varied. A pure oil/water emulsion was also prepared as an oil control reference. Tests were accepted if the percentage of control embryos at 48 h of development was ≥80%, as recommended by standard procedure [24].

The degree of toxicity per treatment was calculated using the standard criteria of evaluation based on the calculation of the percentage of normal versus abnormal embryos for a sample of 100 embryos (in triplicate) by optical microscopy (48 h of development endpoint). Embryonic development was also kept under observation to evaluate the occurrence and timing of several morphological events related to the endoderm, ectoderm, and mesoderm (germ layers) development and differentiation, as reported by Pinsino et al. [25].

### 2.3. Adult Sea Urchin Immune Cell Exposure

Adult sea urchins (*Paracentrotus lividus*) were collected along the northwest coast of Sicily and were acclimated and maintained under controlled conditions of temperature (16 ± 2 °C), pH (8.1 ± 0.1), salinity (38–39%), and density (1.028–1.030 g/cm^3^) in oxygenated ASW (Aqua Ocean Reef Plus Marine Salt, Aquarium Line, Bari, Italy).

Approximately 0.5 mL of coelomic fluid containing freely circulating immune cells were collected from each sea urchin using a 1-mL sterile syringe already containing 0.5 mL of anticoagulant solution, namely Coelomocyte Culture Medium (CCM), composed of 1 M NaCl, 10 mM MgCl_2_, 40 mM Hepes-4-(2-hydroxyethyl)-1-piperazineethanesulfonic acid- and 2 mM EGTA - ethylene glycol tetra-acetic acid- at pH 7.2. After collection, the immune cells were counted in a Fast-Read chamber (Biosigma, Madison, Wisconsin, USA), and morphological analysis of cells was performed using the optical microscope.

After counting, immune cells were plated at a density of 1 × 10^5^ cells/well in a 96-well white, opaque-walled plate (Thermo Fisher Scientific, Waltham, Massachusetts, USA) and exposed to a final volume of 100 μL T-AVO NPs that had been pre-dispersed in stock solutions of pure water (1 and 100 mg/L nominal concentration), sterilized under UV light, and vortexed for 5 min in order to homogenize the dispersions. The NPs were added to each cell culture medium to achieve five different concentrations (0.1, 1, 10, 100, 500 mg/L final concentration). Culturing was performed in the dark at 16 ± 2 °C. Cell viability and cytotoxicity were measured using the RealTime-Glo MT Cell Viability Assay (Promega, Madison, Wisconsin, USA) and the non-lytic CellTox™ Green Cytotoxicity Assay (Promega, USA), respectively, as previously described [26]. Luminescence and fluorescence were detected using a GloMax Discover high-performance Microplate Reader (Promega). All assays involved at least five biological replicates (specimens).

### 2.4. Metabolite Renewal Analysis by Mass Spectrometry in Untargeted Liquid Chromatography

Liquid chromatography-mass spectrometry (LC-MS analysis) was performed to analyze the metabolite profile of sea urchin cells exposed to hydrophilic NPs, according to previously established protocols [27]. Metabolites were isolated in 0.5 mL ice-cold 1% acetic acid water–acetonitrile solution (70:30 *v/v*). Supernatants were recovered in glass inserts for solvent evaporation, dried at 30 °C for approximately 2.5 h (Concentrator plus/Vacufuge^®^ plus, Eppendorf, Juelich, Germany,), re-suspended in 150 μL of H_2_O LC-MS grade, and injected into the UHPLC (Ultra high performance liquid chromatography)–MS system for reversed-phase liquid chromatography (RPLC). Data analysis and isotopic natural abundance correction were performed with MassHunter ProFinder and Mass Pro le Professional software (Agilent, Santa Clara, California, USA).

Samples were analyzed using an UHPLC system (Agilent 1290 Infinity UHPLC system) coupled with a quadrupole time-of-flight hybrid mass spectrometer (Agilent 6550 iFunnel Q-TOF) and equipped with an electrospray Dual JetStream source. The LC reversed phase was separated using an InfintyLab Poroshell 120 PFP-pentafluorophenyl-column (2.1 × 100 mm, 2.7 μm; Agilent Technologies) at a volume of 15 μL, a flow rate of 0.2 mL/min, and a temperature of 35 °C. Both the mobile phase A (100% water) and B (100% acetonitrile) contained 0.1% formic acid. The elution gradient was (1) 0 min, 100% A, (2) 2 min, 100% A, (3) 4 min, 99% A, (4) 10 min, 98% A, (5) 11 min, 70% A, (6) 15 min, 70% A, and (7) 16 min, 100% A followed by 5 min of post-run. Mass spectra were recorded in centroid mode in a mass range from *m*/*z* 60 to 1050 *m*/*z*. The mass spectrometer operated using a capillary voltage of 3.7 kV. Source temperature was set to 285 °C with a 14 L/min drying gas and a nebulizer pressure of 45 psig (pounds per square inch gauge). Fragmentor, skimmer, and octopole voltages were set to 175, 65, and 750 V, respectively.

Active reference mass correction was performed through a second nebulizer using the reference solution (*m*/*z* 112.9855 and 1033.9881) dissolved in the mobile phase 2-propanol-acetonitrile-water (70:20:10 *v*/*v*).

### 2.5. Characterization of the NPs by High-Resolution Scanning Electron Microscopy

The primary particle length of the NPs was measured using high-resolution scanning electron microscopy (HR-SEM). Three to four milligrams of the four pristine TiO_2_ NP powders (T-S, T-Lite, T-AVO, P25 TiO_2_) were dispersed on carbon adhesive tape and analyzed using a Zeiss Gemini500-Field emission SEM. To obtain surface-sensitive imaging at nanoscale resolution, images were recorded at low voltage (1–5 kV) with an in-lens secondary electron detector. Image analysis was then completed in order to distinguish the smallest particulate units constituting the powder grains. The primary particle lengths were obtained from the largest dimension of these smallest units. Fifty particles per sample were measured in order to calculate an average primary particle length.

### 2.6. Characterization of the NP Dispersions by Dynamic Light Scattering

The size distribution of the studied NPs was measured in their respective original dispersing medium (water or oil) using dynamic light scattering. These dispersions corresponded to the NPs as prepared before injection into the exposure media for the in vivo assay. P25 TiO_2_ and T-AVO NPs were each dispersed in pure water at a nominal concentration of 125 mg/L by agitating the appropriate mass of the pristine powders in 5 mL.

The oily dispersions of T-S and T-Lite NPs were prepared by dispersing the UV filters in the commercial cosmetic oil by mechanical agitation at 1000 rpm rotation speed for 10 min, at a nominal NP concentration of 25 g/L using a *Heidolph Hei-Torque* 400 stirrer equipped with a pitcher blade impeller. The hydrophobic UV filters were analyzed in oil at a solid concentration 200 times as high as that for hydrophilic UV filters because in the in vivo assay the oil dispersion was further emulsified by dilution in water but the NP concentration remained locally high in the oil droplets. Moreover, since such complex dispersion/emulsion systems cannot be measured by dynamic light scattering (DLS), only the original oil dispersion was measured here in order to provide insights on the NP aggregation state in the oil droplet.

The size measurements were performed in triplicate at 25 °C with 11 runs per measurement, normal resolution analysis, and 0.01 cumulant fit error tolerance, using a *Zetasizer Nano* (Malvern Instruments, Malvern, UK).

The characterization of the particles and the degree of dispersibility in each pre-dispersant medium are reported in Supplementary Materials.

### 2.7. Statistical Analysis in Biological Assays

Statistical analyses were performed by GraphPad Prism Software 6.01 (USA). Statistical differences among selected groups were estimated by one-way ANOVA (followed by the multiple comparison tests). The p-value lower than 0.05 was deemed statistically significant. Data were expressed as mean ± standard deviation (SD).

## 3. Results and Discussion

### 3.1. Influence of TiO_2_ NP-Based UV Filters on the Growth of Sea Urchin Embryos

The TiO_2_ NPs used as commercial UV filters in sunscreen consist of the rutile lattice form, which is known to be less photoreactive and less toxic than other forms (i.e., anatase, brookite) [16,17]. They are generally coated with a surface passivation layer aimed at suppressing the rutile photocatalytic activity (e.g., Al_2_O_3_, SiO_2_) and a secondary layer added to enhance particle dispersion in the cosmetic formulation (e.g., hydrophobic polydimethylsiloxane, hydrophilic Na-polyacrylate). Recent field studies of recreational bathing waters have evidenced a distinct fate for the hydrophobic compounds released from sunscreen [19,20]). Notably, measured TiO_2_ concentrations were higher (up to 1000x) in the water top surface layer (0.1–0.9 mg/L) than in the water column (0.02–0.05 mg/L).

**Figure 1:**
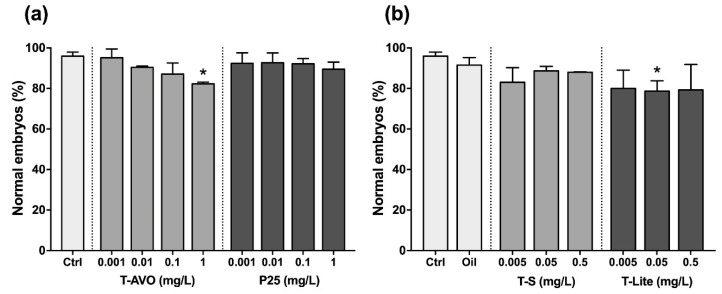
The hydrophilic and hydrophobic TiO_2_ NP-based UV filters at different concentrations and dispersant phases influence the sea urchin embryonic development without producing a relevant developmental impairment. Histograms represent the results expressed as mean percentage (%) of normal embryos ± standard deviation after 48 h of exposure to the (**a**) hydrophilic (T-AVO and P25) and (**b**) hydrophobic (T-S and T-Lite) TiO_2_ NP-based UV filters. * *p* < 0.05.

**Figure 2:**
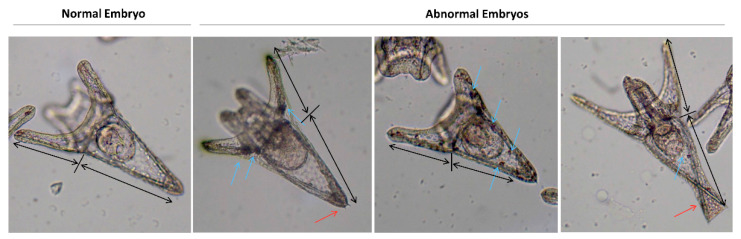
Optical images of representative sea urchin *Paracentrotus lividus* exposed to TiO_2_ NP-based UV filters. Black arrows indicate decrease or increase in arm growth and supporting skeletal rods. Red arrows indicate crossed or separated tips at the hood apex arms. Blue arrows indicate the atypical big cells found in abnormally developed embryos.

Here, we investigated the effects of increasing concentrations of three TiO_2_ NP-based UV filters on the sea urchin *P. lividus* in the embryonic life-cycle stage. Embryos were classified as normal only when they satisfied all the following morphological criteria: (1) Acceptable schedule in reaching the developmental endpoint; (2) dorso/ventral and left/right embryonic axis symmetry; (3) correct differentiation of oral/aboral endoderm and ectoderm; and (4) correct mesenchyme differentiation, distribution pattern, and shape. At the gastrula stage (24 h of development), embryos exposed from fertilization maintained a regular time schedule and proper sites of spicule elongation at all concentrations tested (not shown). In agreement, at the pluteus stage (48 h) TiO_2_ NP-based UV filter-exposed embryos displayed a low number of abnormalities (Figure 1). A slight increase in the incidence of potential morphological abnormalities was observed only in embryos exposed to 1 mg/L of the T-AVO UV filter (hydrophilic) and 0.05 mg/L of the T-Lite UV filter (hydrophobic). Specifically, about 20% of the embryos exhibited problems on arm and skeleton rod development and/or randomly distributed atypical big cells (Figure 2). The most common skeletal malformations observed were: (1) Crossed or separated tips at the hood apex arms (Figure 2, red arrows); (2) asymmetrical arm lengths; and (3) decrease or increase in arm growth and supporting skeletal rods (Figure 2, black arrows, oral and post-oral skeletal rods). Similar skeleton-defective embryos were previously observed in *P. lividus* embryos exposed to other types of NPs, suggesting that skeleton abnormalities could be considered a sensitive target to NP exposure [28,29]. Besides, the formation of big cells or masses (Figure 2, blue arrows) was previously observed in *P. lividus* embryos exposed to PS-NH_2_ NPs and in *Strongylocentrotus droebachiensis* embryos exposed to Ag NPs [25,30]. These cells could be mesodermal cells involved in NP internalization (immune defense). Others have reported that NP internalization can initiate the immune response responsible for the formation of big mesodermal cells [25], such as those seen in Figure 2. However, this hypothesis remains essentially speculative because NP internalization was not verified here. Notably, other plausible explanations cannot yet be excluded, such as the possibility that the formation of big cells or masses may be caused by other compounds and not by NP internalization. However, similar effects were not observed in embryos exposed to the pure sunscreen oil phase only (Figure 1b), indicating that the oil phase compounds could not be incriminated in the formation of big cells or masses. Further studies are needed to clarify the underlying mechanism.

In addition, we would like to point out that the morphological abnormalities observed in the sea urchin embryos exposed to NPs may not even be of ecological significance. Indeed, there was no evidence that these abnormalities reduced larvae survival ability, thus endangering a whole of one population.

Li et al. proposed a model of interaction between NPs and lipid layers of the plasma membrane based on the hydrophobic/hydrophilic nature of particles [31]. Based on the model, they suggested that hydrophobic NPs, which are thermodynamically stable around the core of a bilayer hydrophobic membrane, lead to lipid molecule deformation and distribution, whereas hydrophilic NPs that adsorb on the membrane surface (ready to be phagocytized) enter the hydrophobic core of the membrane, maintaining it intact. The primary particle shape also influences and modulates the particle behavior in a medium and its subsequent biological effect on a living system. Brown et al. argued that rod-shaped NPs could interact more strongly with biological systems than round-shaped NPs because the van der Waals interaction forces in lengthwise-oriented NPs increase proportionally to their length, typically reaching values several orders of magnitude above that of spheres [32]. Based on this theory, the elongated shape of T-AVO NPs compared to the P25 TiO_2_ NPs (Figure S1, Supplementary Materials) would facilitate the interaction of particles with the membranes.

It is unclear how the dispersion states of the different UV filters measured here in the aqueous or oil cosmetic medium (Table S1, Supplementary Materials) are altered after dispersion and dilution in the aqueous culture medium. Aggregation and sedimentation will surely occur in artificial seawater for the hydrophilic UV filters pre-dispersed in water because salt-induced aggregation is a well-known effect in such a system [33]. On the contrary, further aggregation is not expected for the hydrophobic UV filters, as they should remain in the oil phase during the exposure. It is important to mention that hydrophobic filters trapped in the surface microlayer may not be bioavailable to the organisms living in suspension because the oil phase does not naturally mix with water. However, the surfactant molecules used in the cosmetic formulation may favor the mixing of the aqueous and oil phases and might play a determining role here (Table 2). For example, it is known that the octyldodecyl xyloside has high affinity for T-Lite NP surface [13]. Thus, we can reasonably assume that once washed of the consumer’s skin, these molecules can modify the NP fate in the aquatic environment and their subsequent bioavailability for the marine organisms.

Overall, our results confirmed that the tested hydrophilic and hydrophobic TiO_2_ NP-based sunscreen filters do not elicit significant harmful effects on sea urchin embryonic development. Of note, we were not fully satisfied with the efficacy of our evaluation assay with the hydrophobic UV filters pre-dispersed in the oil. Increasing the NP concentration in the oil implies an increase in viscosity. This makes the approach difficult to apply at high concentrations, as well as in tests under static conditions (e.g., primary immune cell culture). For this reason, only the hydrophilic TiO_2_ NPs were used in subsequent immune cell-based assays.

### 3.2. Sea Urchin Adult Immune Cells: Health State and Metabolic Typing under Hydrophilic TiO_2_ NPs

**Figure 3:**
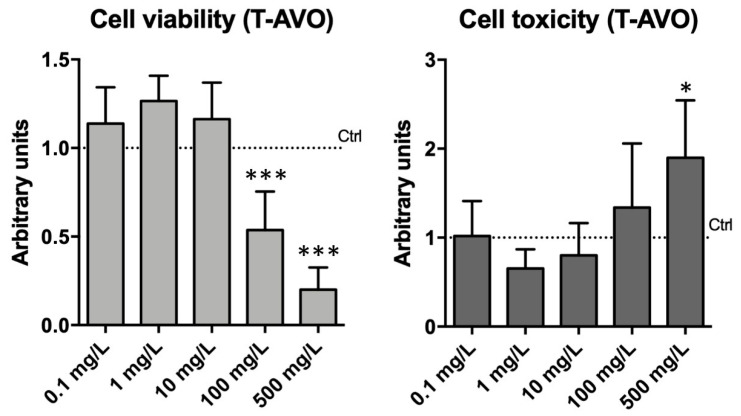
Impact of T-AVO UV filters on the sea urchin immune cell viability and toxicity. Real-time viability over two days of continuous monitoring, of which one measurement point (48) is shown (0.1, 1, 10, 100, 500 mg/L final concentration). The highest doses (100 and 500 mg/L) provoked decreases in cell viability and increase in cell toxicity. Levels are expressed in arbitrary units as fold increase or decrease compared to controls assumed as 1 (dot line). Data are reported as the mean ± SD; stars (*) indicate significant differences among groups (* *p* < 0.05; *** *p* < 0.001).

An ideal sunscreen active agent should remain localized close to the skin surface without penetrating into the deeper layers because by entering in the systemic circulation it could stimulate immune reactions. The sea urchin *P. lividus* can function as a proxy for humans for in vitro immunological studies [34]. Thus, to focus on the sea urchin immunological tolerance to the silica-coated TiO_2_ NP-based UV filters we assessed the viability and cytotoxicity of the exposed immune cells to the hydrophilic T-AVO NPs for 48 h and we characterized their metabolic profile after 72 h of exposure. Cell viability and cytotoxicity were monitored and measured in real time for cells exposed to increasing concentrations of T-AVO NPs (0.1, 1, 10, 100, 500 mg/L). Only the measurements at 48 h are shown (Figure 3). The highest concentrations (100, 500 mg/L) were used as positive controls to demonstrate the appropriateness of the procedures.

Our results confirmed those obtained from the sea urchin embryonic development assay, where no significant toxic effects were found at concentrations from 0.1 to 10 mg/L. At the two highest concentrations of exposure (100 and 500 mg/L), a significant decrease in viability and a high cell toxicity were measured, as expected. At these concentrations, faster NP aggregation likely led to particle destabilization and sedimentation, which, in turn, affected cellular viability due to the aggregate–cell physical contact. For samples exposed to 0.1–10 mg/L T-AVO, the RealTime-Glo MT cell viability assay indicated an increase (not statistically significant) in cell viability/metabolic activity compared to unexposed controls. Notably, the chemistry assay is based on the reducing potential of the cell, which is a known metabolic marker of cell viability. This result may be due to a hysteresis response usually observed under drug administration, in which the effect of a drug declines despite its continued presence (drug tolerance) [35]. Studies elucidating metabolic profiles of immune cells exposed to T-AVO were, therefore, carried out to confirm this increased metabolic activity, as reported below.

**Figure 4:**
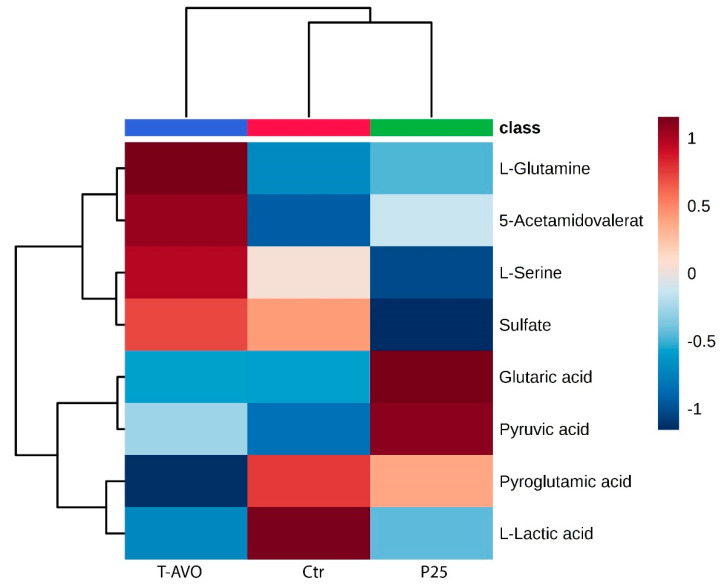
Sea urchin immune cell metabolic profile under hydrophilic nano-TiO_2_-based UV filters. Untargeted metabolic profiling of T-AVO and P25 exposed for one day at 2 mg/L and for two days at 1 mg/L and unexposed immune cells (Ctr) for 72 h. Hierarchical clustering heatmaps display significantly (*p* ≤ 0.05) different intracellular metabolites by LC-MS. Metabolic typing was performed on *P. lividus* primary immune cell cultures obtained from five individual donors.

Immune functions are bio-energetically expensive, requiring accurate management of metabolites coordinated by intracellular and extracellular signals, which direct the uptake, storage, and utilization of substrates (e.g., glucose, amino acids, fatty acids). In turn, metabolites renew and control immune responses [36]. In order to obtain integrated data on the sea urchin immune metabolic state during exposure to hydrophilic TiO_2_ NP-based UV filters and their related tolerance state, we characterized the metabolic profile of cells exposed to T-AVO and compared it with the profiles of cells exposed to P25 and unexposed cells (72 h in culture) (Figure 4). To this purpose we used an initial higher dose of particles (2 mg/L, loading dose) to achieve a lower maintenance dose (1 mg/L).

Metabolite profiling identified level changes of only eight metabolites among groups (Group 1: T-AVO 1-day exposure at 2 mg L^−1^ followed by 2-days exposure at 1 mg L^−1^; Group 2: Control, 3 days; Group 3: P25 1-day exposure at 2 mg/L followed by 2-days exposure at 1 mg/L), including proteinogenic amino acids (serine, glutamine), amino acid derivatives (L-pyroglutamic acid), organic acids (L-lactic acid, glutaric acid, pyruvic acid), sulfate metabolites (sulfate), and acetamides (acetamidovalerate). Sea urchin T-AVO-responsive metabolites predominantly involved in mediating inflammatory signals and phagocytosis were found. Specifically, glutamine and acetamidovalerate were significantly increased compared to unexposed controls, while L-pyroglutamic acid and L-lactic acid were significantly decreased. It is well known that to re-establish normal cellular and molecular function, immune cells have higher glutamine needs during inflammatory states [37]. Notably, acetamides are known to relieve inflammatory events; in fact, they are used as chemotherapeutic agents for inflammation-associated cancers [38].

Lactic acid is produced in high amounts by innate immune cells during inflammatory activation (anaerobic glycolysis product). A reduction in its amount may be translated into a negative feedback signal to silence or attenuate inflammatory responses [39].

Increased levels of pyroglutamic acid (also called 5-oxoproline) are able to promote lipid and protein oxidation and to enhance hydrogen peroxide content, thus promoting oxidative stress [40]. Consequently, the significantly decreased pyroglutamic acid levels observed under T-AVO exposure may be considered a signal of an increased antioxidant metabolic activity. Although slight, L-serine and pyruvic acid levels were also increased compared to those in unexposed cells. Serine racemase enzyme catalyzes the α, β elimination of water from L-serine to produce pyruvate and ammonia [41]. Notably, L-serine metabolism is required for fueling one-carbon metabolism and nucleotide biosynthesis, and it is known to lower the inflammatory responses in mice during infection [42].

The P25 TiO_2_ bare NPs were used here as a negative control for the silica-functionalized T-AVO. In our recent studies we demonstrated that under TiO_2_ NP exposure (1 mg/L TiO_2_ NPs, 24 h endpoint) the innate sea urchin immune system is able to control inflammatory signaling, excite antioxidant metabolic activity, and acquire immunological tolerance [27,43]. Notably, sea urchin immune system metabolic typing under P25 exposure for 72 h (one day of exposure at 2 mg/L followed by two days of exposure at 1 mg/L) highlighted a different scenario compared to the respective T-AVO and control groups. L-glutamine, acetamidovalerate, and pyriglutamic acid levels all presented a trend similar to controls, while lactic acid levels were similar to T-AVO-exposed cells and pyruvic acid levels were considerably increased. Interestingly, L-serine, sulfate, and glutaric acid levels presented a trend completely different to both T-AVO and control groups. Specifically, L-serine and sulfate were reduced compared to controls, while glutaric acid levels increased, highlighting an ongoing inflammatory state. These findings show the superior immune compatibility of T-AVO compared to P25 when particles stay in contact with the sea urchin immune cells for 72 h (one day of exposure at 2 mg/L, and two days of exposure at the half dose). Based on the notion that the silica coating of commercial TiO_2_ NP-based UV filters undergoes a fast degradation once released into aquatic media (88–98% silica removal within 96 h) [44], we speculated that the T-AVO in contact with cells after 72 h of exposure consisted of mainly pure rutile. Worth noting is the fact that not only the shape but also the crystal composition of TiO_2_ particles (e.g., T-AVO: Pure rutile; P25: Anatase:Rutile) makes their use more or less safe, as documented in the literature [45].

**Figure 5:**
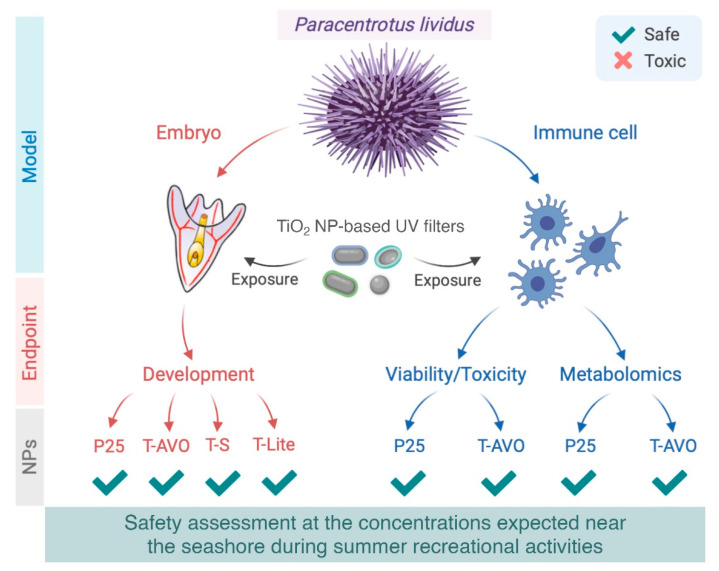
Schematic illustration of the results obtained from the evaluation of the TiO_2_ NP-based sunscreen UV filters on the development and the immunological state of the sea urchin *Paracentrotus lividus*. Models: Sea urchin embryos and adult *P. lividus* immune cells in vitro. Endpoints: Sea urchin embryos at the pluteus stage (48 h), immune cell viability/toxicity (48 h), and metabolomics (72 h). Commercial nanoparticulate UV filters with three surface coatings: T-S, T-Lite, and T-AVO tested on embryonic development and T-AVO on immune cells. P25 was used as an uncoated nano-TiO_2_ reference. The results presented here highlight the biosafety of TiO_2_ NP-based UV filters on sea urchin, and the importance of developing safer-by-design sunscreens and evaluating the associated risk. Created by Andi Alijagic using BioRender.com.

Overall, our results demonstrate that the commercial TiO_2_ NP-based UV filters tested in this work do not show any significant harmful impact toward the development or immunity of the sea urchin (*P. lividus*) at NP concentrations expected near the seashore during summer recreational activities. A summary view of the experimental setup and the results is schematized in Figure 5.

These results are in accordance with the majority of risk assessment studies on different marine organisms using pure rutile or anatase NPs that are summarized in the most relevant reviews [3,46]. The different coatings on the commercial UV filters, together with particle shape and the original dispersant phase, slightly modulate the effects toward embryo development but without causing a relevant developmental impairment. In this context, the particle shape of hydrophilic NPs pre-dispersed in water, predominantly influences the interaction with sea urchin embryos compared with other physical features such as primary particle size or aggregation state. Particularly, the elongated, rod-shaped T-AVO NPs slightly impacted the development compared to the more spherical P25 NPs. The effects of hydrophobic UV filters are, instead, in line with their respective dispersion capacity in the former sunscreen oily dispersant medium, which is directly related to their different particle external coatings. The more finely dispersed T-Lite NPs showed a few visible effects compared to the T-S NPs, probably because of specific interactions with the octyldodecyl xyloside surfactant present in the sunscreen oily medium, which would likely ease its transportation inside the embryonic culture medium.

Viability and toxicity tests on immune cells showed no toxicity of T-AVO NPs at concentrations consistent with that of the cosmetic life cycle. Furthermore, metabolic profile characterization performed on P25 and T-AVO NPs showed that the T-AVO NPs have a superior immune compatibility to that of the P25 after 72 h of interaction with sea urchin immune cells, ultimately supporting the safety of this type of commercial TiO_2_-based UV filter on the immunological state of the sea urchin.

Notably, the application of metabolomics in the environmental field is relatively new, but the technique is actively attracting the attention of the scientific community because results obtained successfully describe a “picture” of the biochemistry of an organism, cell, or tissue at any one time [47].

## 4. Conclusions

Our findings underline the importance of developing sustainable sunscreen formulations based on nanoparticulate UV filters, rather than the bare NP counterpart, to minimize the environmental risk posed by sunscreen products. Further studies with a similar approach need to be performed in the future, on different biological models and with different experimental conditions, in order to fully confirm the safety of these nano-products. Primary cell cultures accurately represent the biological microenvironment in which cells reside in tissues, as cell–cell signaling remains preserved [48]. However, the next step should be to study the immunological state of the sea urchins and other organisms that live in the beaches during recreational activity (field study).

## Figures and Tables

**Table 1:** TiO_2_ NP powder commercial names, supplier name, chemical composition, and primary particle size declared by the suppliers.

Powder Name	Powder Supplier	Chemical Composition	Primary Particle Size
Eusolex^®^ T-S	Merck	TiO_2_ (73–79%)/Al_2_O_3_/stearic acid	ND
T-Lite^TM^ SF	BASF	TiO_2_ (79–89%)/Al(OH)_3_/polydimethylsiloxane	14–16 nm
Eusolex^®^ T-AVO	Merck	TiO_2_ (79.6%)/SiO_2_	ND
P25 TiO_2_	Evonik Degussa	TiO_2_ (Anatase/Rutile 4:1)	~21 nm

* ND = not declared.

**Table 2:** Components constituting the typical sunscreen oil phase used in this work: Product names, supplier names, function of each product, and related chemical composition.

Product Name	Supplier	Function	Chemical Composition
Tegosoft P	Evonik	Emollient oil	Isopropyl palmitate
Cetiol LC	BASF	Emollient oil	Coco-Caprylate/Caprate
Easynov	SEPPIC	Emulsifying agent	Octydodecanol; Octyldodecyl xyloside; PEG-30 Dipolyhydroxystearate

## Supplementary Materials

The following are available online at https://www.mdpi.com/2079-4991/10/11/2102/s1, Figure S1: High-resolution scanning electron microscopy (HR-SEM) analysis of the pristine TiO_2_-based NPs. Table S1: Comparison of primary particle size and hydrodynamic aggregate size of the TiO_2_NPs.
