# Supplementary material for: Safety Evaluation of TiO2 Nanoparticle-Based Sunscreen UV Filters on the Development and the Immunological State of the Sea Urchin Paracentrotus lividus
Source: Nanomaterials (Basel). 2020 Oct 23;10(11):2102. doi: 10.3390/nano10112102 (PMC7690680; doi:10.3390/nano10112102)
Supplement: Supplementary file 1 [file nanomaterials-10-02102-s001.pdf]

nanomaterials

Supporting Information

Safety Evaluation of TiO_2 Nanoparticle-Based Sunscreen UV Filters on the Development and the Immunological State of the Sea Urchin *Paracentrotus lividus*

Riccardo Catalano [1], Jérôme Labille [1,†], Daniela Gaglio [2,3], Andi Alijagic [4], Elisabetta Napodano [3], Danielle Slomberg [1], Andrea Campos [5] and Annalisa Pinsino [4,*,†]

[1] Aix Marseille University, CNRS, IRD, INRAE, Coll France, CEREGE, 13545 Aix-en-Provence, France; catalano@cerege.fr (R.C.); labille@cerege.fr (J.L.); Slomberg@cerege.fr (D.S.)
[2] Consiglio Nazionale delle Ricerche, Istituto di Bioimmagini e Fisiologia Molecolare (IBFM), 20090 Segrate, MI, Italy; daniela.gaglio@ibfm.cnr.it
[3] SYSBIO.IT, Centre of Systems Biology, University of Milano-Bicocca, 20126 Milano, Italy; elisabetta.napodano@unimib.it
[4] Consiglio Nazionale delle Ricerche, Istituto per la Ricerca e l'Innovazione Biomedica (IRIB), 90146 Palermo, Italy; andialijagic@gmail.com; annalisa.pinsino@irib.cnr.it
[5] Aix Marseille Université, CNRS, Centrale Marseille, FSCM, CP2M, 13397 Marseille, France; andrea.campos@univ-amu.fr
* Correspondence: annalisa.pinsino@irib.cnr.it or annalisa.pinsino@cnr.itd
† These authors share senior authorship.

Characterization of the TiO_2 NPs Constituting the UV Filters and Degree of Dispersibility in the Pre-dispersant Mediums

The primary particle size corresponds to the larger dimension of the elementary TiO_2 crystallites constituting the UV filter. They are generally assembled in larger aggregates existing in the dry powder form. Those aggregates can be partly fractionated when the UV filters are dispersed in a liquid. Scanning Electron Microscopy was used to determine the primary particle size of the TiO_2 NP powder (not declared by the suppliers) used as UV filters for sunscreen formulation, and compared to P25 TiO_2 NPs used as non-coated nano-TiO_2 reference. Results are shown in Figure S1, and the sizes (larger dimension) obtained from the SEM images are reported as nm in Table S1. The P25 TiO_2 NPs showed a non-uniform spherical morphology (Figure S1a) with a diameter of 22.7 ± 4.3 nm, in accordance with what declared by the supplier (compare Table 1 and Table 1S). The TiO_2 NPs coated with a hydrophilic layer of silica (T-AVO) showed a more spherical elongated shape than P25 TiO_2 NPs (Figure S1b) and a primary particle size of 51.0 ± 10.6 nm, whereas both TiO_2NPs coated with hydrophobic layers, respectively dimethicone (T-Lite) and stearic acid (T-S), showed a rod-like shape (Figure S1c, S1d), and a primary particle sizes of 58.3 ± 10.7 and 64.4 ± 11.4.

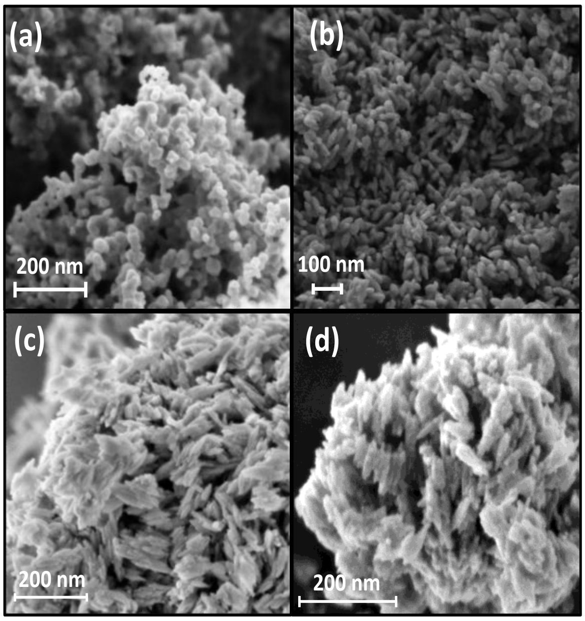

Figure S1. High-resolution scanning electron microscopy (HR-SEM) analysis of the pristine TiO_2-based NPs. (**a**) P25 TiO_2; (**b**) T-AVO, (**c**) T-Lite and (**d**) T-S.

The dispersion state of the TiO_2 NPs in the water and in the cosmetic oil was evaluated using Dynamic Light Scattering (DLS). The average size measurement obtained for each NPs is reported in intensity weight (Table S1**)**. The T-AVO average size in water was 1543.7 ± 208.6 nm, three times larger than P25 TiO_2 NPs (552.6 ± 5.8 nm, average size). This could be due to stronger interparticle attractions between the T-AVO NPs than between the P25 NPs, already existing in the original powder form. The spherical/elongated shape of the former may favour this aggregation, due to a larger surface are of interaction between the NPs.

Table S1. Comparison of primary particle size and hydrodynamic aggregate size of the TiO_2 NPs. Primary particle size measurements of each dry powder was determined by SEM. Hydrodynamic size measurement was determined by DLS after dispersion in water (P25 TiO_2 and T-AVO NPs, 125 mg/L) or cosmetic oil (T-S and T-Lite NPs, 25 g/L).

TiO_2 NPs	Primary particle size (nm)	Hydrodynamic size (nm)
P25 TiO_2	22.7 ± 4.3	552.6 ± 5.8
T-AVO	51.0 ± 10.6	1543.7 ± 208.6
T-Lite	58.3 ± 10.7	173.9 ± 63.5
T-S	64.4 ± 11.4	857.3 ± 131.7

The average T-Lite NPs size in the cosmetic oil was 173.9 ± 63.5, whereas the T-S NPs resulted in an average size five times larger, indicating a more pronounced aggregation (857.3 ± 131.7 nm, average size). This results from the respective affinities of the UV filter surface coatings with the oil components. The higher affinity of the T-Lite with the oil favours its dispersion, while the lower affinity of the T-S with the oil favours the homo-aggregation of the particles that minimize the surface area of interaction with the solvent [1].

It is unclear how the dispersion states of the different UV filters measured here in the aqueous or oil cosmetic medium are altered after dispersion and dilution in the culture medium. NP fate is then characterized by co-evolving size and concentration of the suspended NPs. Such mechanism is challenging to track *in situ* in the exposure medium because the NP concentration decreases close to zero while the composition of the complex medium can lead to multiple scenarios of interaction such as hetero-aggregation [2,3]. This was not investigated here. However, aggregation and sedimentation

will surely occur in artificial seawater for the hydrophilic P25 TiO_2 and T-AVO NPs pre-dispersed in water because salt-induced aggregation is a well-known effect in such system [4]. The sea urchin coelomic fluid (blood equivalent) resembles natural seawater and contains functional biomacromolecules that interact with the TiO_2 NPs to form a biomolecule coating, which may lead to a higher stability in suspension or an increased aggregation [5].

The T-S and T-Lite hydrophobic filters remained mostly concentrated in the oil phase over the duration of the exposure, floating at the culture medium surface and limiting the interaction of the NPs with embryos to the area close to the oil/water interface. However, the migration of the NPs from the oil to the water may be facilitated by the amphiphilic components of the cosmetic oil, e.g. surfactant molecules. For T-S and T-Lite UV filters, further aggregation is not expected, as we assume that these hydrophobic filters remain in the oil phase during the exposure without entering in contact with the aqueous culture medium. For these reasons, we assume that the average sizes measured by DLS and reported for the hydrophobic UV filters dispersed in cosmetic oil are mostly preserved during the embryo exposition, while those returned with the hydrophilic UV filters pre-dispersed in water are totally different, because particle aggregation is expected.

References

1. Catalano, R.; Masiona, A.; Ziarelli, F.; Slomberg, D.; Laisney, J.; Unrine, J.M.; Campos, C.; Labille, J. Optimizing the dispersion of nanoparticulate TiO2-based UV filters in a non-polar medium used in sunscreen formulations – The roles of surfactants and particle coatings. *Colloid Surf. A.* **2020**, *599*, 124792.
2. Labille, J.; Feng, J.; Botta, C.; Borschneck, D.; Sammut, M.; Cabie, M.; Auffan, M.; Rose, J.; Bottero, J.-Y. Aging of TiO2 Nanocomposites Used in Sunscreen. Dispersion and Fate of the Degradation Products in Aqueous Environment. *Environ. Pollut.* **2010**, *158*, 3482–3489.
3. Domingos, R. F.; Baalousha, M. A.; Ju-Nam, Y.; Reid, M. M.; Tufenkji, N.; Lead, J. R.; Leppard, G. G.; Wilkinson, K. J. Characterizing Manufactured Nanoparticles in the Environment: Multimethod Determination of Particle Sizes. *Environ. Sci. Technol.* **2009**, *43*, 7277–7284.
4. Labille, J.; Brant, J. Stability of Nanoparticles in Water. *Nanomed.* **2010**, *5*, 985–998.
5. Alijagic, A.; Benada, O.; Kofroňová, O.; Cigna, D.; Pinsino, A. Sea Urchin Extracellular Proteins Design a Complex Protein Corona on Titanium Dioxide Nanoparticle Surface Influencing Immune Cell Behavior. Front. Immunol. **2019**, *10*, 2261.
